# Predictors of historical change in drug treatment coverage among people who inject drugs in 90 large metropolitan areas in the USA, 1993–2007

**DOI:** 10.1186/s13011-019-0235-0

**Published:** 2020-01-09

**Authors:** Barbara Tempalski, Leslie D. Williams, Brooke S. West, Hannah L. F. Cooper, Stephanie Beane, Umedjon Ibragimov, Samuel R. Friedman

**Affiliations:** 1 Institute for Infectious Disease Research, NDRI, Inc., 71 West 23rd Street, 4th Fl, New York, NY 10010 USA; 2 School of Social Work, Columbia University, New York, NY USA; 3 Rollins School of Public Health, Emory University, Atlanta, GA USA; 4 Department of Population Health, New York University, New York, NY USA; 5 Bloomberg School of Public Health, Johns Hopkins University, Baltimore, MD USA

**Keywords:** Injection drug use, Predictors, Drug treatment coverage, Longitudinal, Mixed-effects multivariate models, Metropolitan areas, Drug policy, Theory of community action

## Abstract

**Background:**

Adequate access to effective treatment and medication assisted therapies for opioid dependence has led to improved antiretroviral therapy adherence and decreases in morbidity among people who inject drugs (PWID), and can also address a broad range of social and public health problems. However, even with the success of syringe service programs and opioid substitution programs in European countries (and others) the US remains historically low in terms of coverage and access with regard to these programs. This manuscript investigates predictors of historical change in drug treatment coverage for PWID in 90 US metropolitan statistical areas (MSAs) during 1993–2007, a period in which, overall coverage did not change.

**Methods:**

Drug treatment coverage was measured as the number of PWID in drug treatment, as calculated by treatment entry and census data, divided by numbers of PWID in each MSA. Variables suggested by the Theory of Community Action (i.e., need, resource availability, institutional opposition, organized support, and service symbiosis) were analyzed using mixed-effects multivariate models within dependent variables lagged in time to study predictors of later change in coverage.

**Results:**

Mean coverage was low in 1993 (6.7%; SD 3.7), and did not increase by 2007 (6.4%; SD 4.5). Multivariate results indicate that increases in baseline unemployment rate (β = 0.312; *pseudo-p* < 0.0002) predict significantly higher treatment coverage; baseline poverty rate (β = − 0.486; *pseudo-p* < 0.0001), and baseline size of public health and social work workforce (β = 0.425; *pseudo-p* < 0.0001) were predictors of later mean coverage levels, and baseline HIV prevalence among PWID predicted variation in treatment coverage trajectories over time (baseline HIV * Time: β = 0.039; *pseudo-p* < 0.001). Finally, increases in black/white poverty disparity from baseline predicted significantly higher treatment coverage in MSAs (β = 1.269; *pseudo-p* < 0.0001).

**Conclusions:**

While harm reduction programs have historically been contested and difficult to implement in many US communities, and despite efforts to increase treatment coverage for PWID, coverage has not increased. Contrary to our hypothesis, epidemiologic need, seems not to be associated with change in treatment coverage over time. Resource availability and institutional opposition are important predictors of change over time in coverage. These findings suggest that new ways have to be found to increase drug treatment coverage in spite of economic changes and belt-tightening policy changes that will make this difficult.

## Introduction

A key pillar of public health planning is that the magnitude of a response needs to match the magnitude of a problem. In the United States (US) and its large metropolitan areas, however, despite repeated calls for expansion of drug treatment, treatment coverage for people who inject drugs did not increase overall during the period 1993–2007 and continues to fall far short of need [1–4]. 

A second pillar of public health is that the distribution of programs across geographic areas should reflect the geographic distribution of need for that program. However, research suggests that local need for a wide variety of different types of programs fails to predict local program presence or coverage. Friedman and colleagues, for example, have found that syringe service programs (SSPs) and drug treatment coverage for people who inject drugs (PWID) varies greatly across metropolitan statistical areas (MSAs), and that local *need* does not predict these variations [4–7]. Rather, the political influence of men who have sex with men (MSM) is associated with more program coverage for PWID, and government budget limitations (i.e., long term debt per capita) predict less coverage [4–6]. Need also does not predict the presence or coverage of other health and social service programs (e.g., programs against drunk drivers or smoking) [8–16]. Such research suggests that the presence and coverage of public and social service programs is influenced by local policy environment, and not by local need. In order to adequately address the elimination of new HIV transmissions and response to epidemiological need in low resource areas, community-based organizations need the ability to rapidly change and assemble new prevention services to meet the challenge of changing epidemiology, population demographics, and advances in technology, or policy/political imperatives.

Low treatment coverage for PWID may produce a high cost to society in terms of the spread of HIV, hepatitis B and C and other infectious diseases among injectors, their partners, and the broader community [17, 18]. Evidence-based drug treatment such as methadone maintenance therapy and buprenorphine can address a broad range of social and public health problem valued in communities affected by PWID [18, 19]. Adequate access to effective treatment and medication assisted therapies for opioid dependence has led to a decrease in HIV transmission, improved ART adherence and decrease in morbidity and mortality not only for opioid overdose but also HIV/AIDS related disease [18, 20–24]. Research is needed to address what policy and structural changes affect variations and changes in treatment coverage - and, in particular, what combinations of factors lead to increases in treatment coverage.

A previous paper showed that the magnitude of drug treatment coverage for people who inject drugs did not increase in large US metropolitan areas over the 15 years, 1993–2007 [1–4]. Here, we study whether the increases and decreases in coverage among various US metropolitan areas seemed to respond to the need in those metropolitan areas. Thus, this paper presents historic trends and predictors of change in drug treatment coverage for PWID in 90 US MSAs during 1993–2007. Our drug treatment sample for calculating treatment coverage includes clients enrolled in residential or ambulatory inpatient/outpatient care, detoxification services, and methadone maintenance therapy at publicly- and privately-funded substance abuse agencies receiving public funds. Coverage was measured as the number of PWID in drug treatment, calculated by using data from the Substance Abuse and Mental Health Service Administration, divided by numbers of PWID in each MSA.

Additionally, we present theory-based predictors of metropolitan treatment coverage rates as a function of program *need, resource availability, institutional opposition, organized support, and service symbiosis,* factors which may contribute to greater coverage of drug treatment programs and coverage. The present study extends our research on the predictors of drug treatment coverage for PWID to include longitudinal data. Understanding which metropolitan characteristics are related to changes in treatment coverage can assist public health policy planners, treatment providers and grassroots organizations in improving access to treatment and in facilitating its spread in areas of need.

## Theoretical framework and selection of predictors

**Fig. 1:**
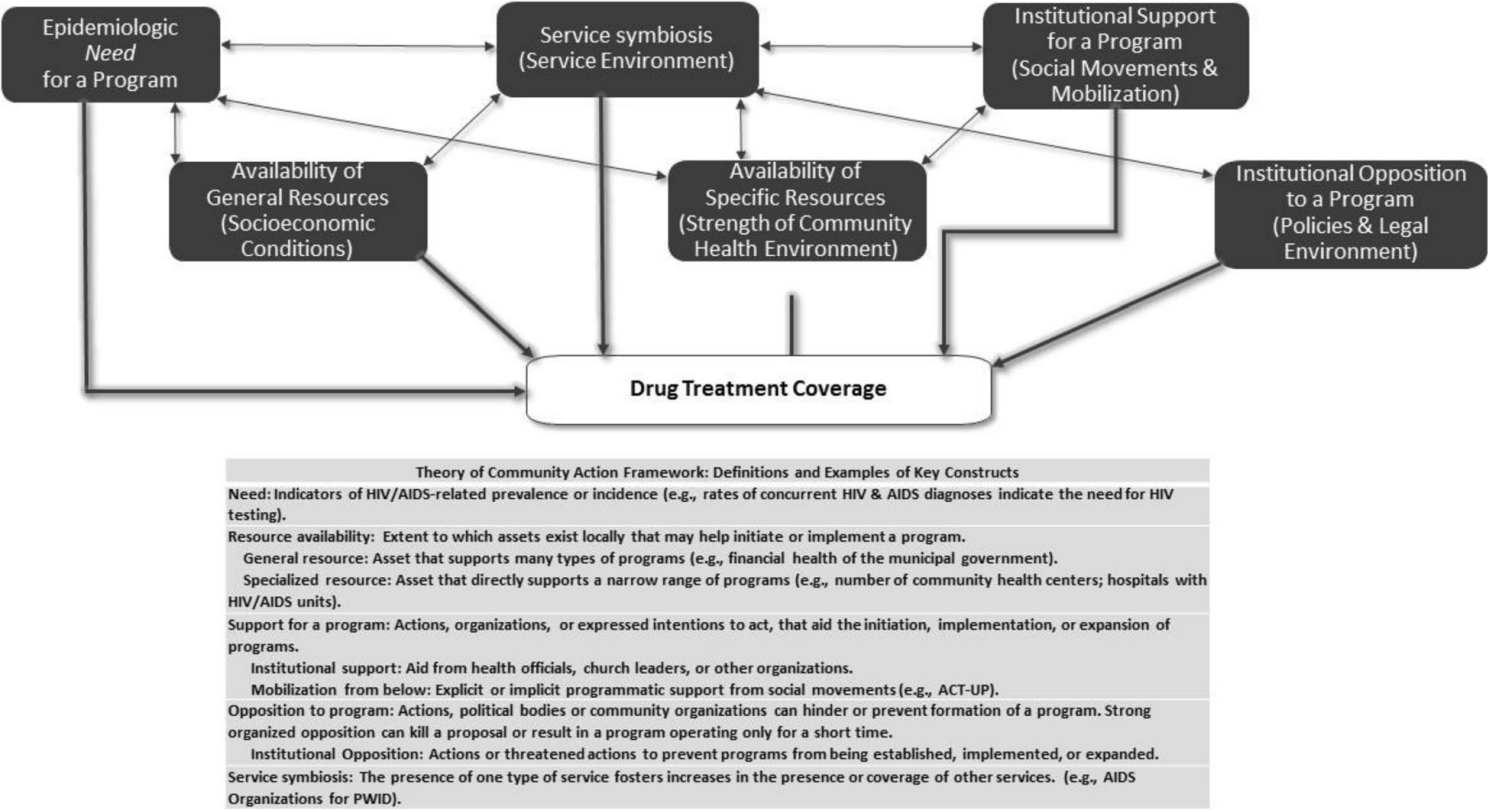
Theory of Community Action Framework and Contextual Factors Predicting Program Presence

Here, we present a theoretical framework for predicting program presence using the “theory of community action” (TCA). Figure 1 displays the conceptual model of TCA. This framework utilizes concepts from urban studies [25–27] social movement theory [28–30] and diffusion of innovations theory [31–34]. It has been used to identify a variety of place characteristics that are likely to affect the extent to which a community carries out and sustains an action. Rosser & Horvath for example, found that successful rural HIV prevention was less likely in states with more religious and Evangelical Protestant adherents and more successful in states with more “gay community” infrastructure [35]; and providing condom distribution interventions or programs [20, 36], counseling and testing services [37, 38], or specialized programs to reduce opiate misuse [3–7, 39, 40] or treatment for people who are mentally ill [41, 42]. As previously conceived [3–7] this framework emphasizes five types of place characteristics as important to the distribution and implementation of institutionalized programs in cities or MSAs. We define each of these domains below:

In this analysis, *need* refers to rates of epidemiologic factors related to drug use, injection drug use, and HIV (e.g., such as PWID AIDS cases per 10,000 population). The idea that *need* leads to response suggests that MSAs with greater epidemiologic need will respond to such need by providing more access to services.

*Resource availability* refers to the extent to which assets that exist locally are useful in initiating and implementing a program. Higher levels of local resource availability are hypothesized to be associated with higher rates of service provision. Local resources can be categorized as either general or specialized. *General resources* might support a wide range of activities [3–7] and may be positively associated with a number of social and health programs that may have an impact (either directly or indirectly) on programs for PWID. General resources may include economic prosperity in an MSA, the financial health of local governments, and the percent of the local population who are college educated [5, 6]. *Specialized resources* support the development of only a narrow range of programs, such as breast cancer programming, mental health programs, or health insurance for children [8, 12, 13]. For the purposes of this research, specialized resources will be defined as resources that support HIV and AIDS programs for PWID. These resources include the skilled labor force coming from medical and public health schools; hospitals with special HIV/AIDS units and staff; and emergency substance abuse services, including HIV/AIDS counselors. Levels of specialized resources are expected to affect key outcomes and the presence of a substance abuse treatment program. In addition, general resources such as a larger, more educated labor pool may increase the efficiency of service provision [5, 6, 37].

*Organized and potential institutional opposition to programs* (even presumed opposition) can hinder or prevent formation of a program. Strong organized opposition can kill a proposal or result in a program operating only for a short time. Drug treatment services for PWID in US communities remain controversial and face ongoing obstacles from law enforcement and local communities, including ‘not-in-my-backyard’ community opposition [37, 39, 43, 44]. As such, organized opposition is hypothesized by the present research to predict declines in, or lower levels of, drug treatment coverage. *Institutional opposition to programs* may include “legal repressiveness” as a competing strategy for maintaining social order [39, 43–47] by controlling drug use. Institutional opposition to programs may also include the mixing of drug policy and racial subordination by the enactment of harsher penalties for possession of drugs among ethnic communities [43–47].

Previous research has shown that racial economic and political structures may adversely affect the health of a variety of racial/ethnic groups as well as reducing the resources and services available to them, including local services for drug users [37, 39, 40, 43–48]. Racial structures such as greater inequality between racial groups, for example, might contribute to, or be reflective of, local environments that are more institutionally oppressive to some groups (i.e. racial minorities), and therefore less likely to provide sufficient resources and services that meet the needs of these groups. Thus, we hypothesize that structural conditions of racial/ethnic inequality may be associated with stronger opposition to drug treatment, and thus may be associated with less drug treatment coverage.

The strength of institutional opposition has often contributed to greater delays in implementation or total lack of implementation of HIV prevention programs for PWID and other public health intervention programs for drug users in U.S. cities and metropolitan areas [37–40, 43, 44, 47, 48]. Research by Shaw, for example (2006) [40] found that strong community opposition, including negative and stigmatizing attitudes toward drug users, was crucial in the failure to establish public harm reduction programs in Springfield, MA. Similar results suggesting that opposition can weaken or prevent harm reduction programs has been found by Tempalski et al., [6, 43, 44]; Downing et al., [37] and Des Jarlais et al., [47, 48]. Likewise, opposition has been identified as a hindrance to services and programs for the homeless, to housing programs for the mentally ill, to half- way houses for people living with AIDS, and to health-related facilities for persons with AIDS [14, 15, 49–54].

*Organized and potential institutional support for programs.* Theoretically converse to the notion of opposition, organized support for programs is hypothesized by the present research to predict increases in, or higher levels of, drug treatment coverage. Indicators of organized or potential support can come from the presence of outreach efforts, ACT UP chapters, local American Public Health Association units, or Planned Parenthood strength. Organized or potential support for a program can determine its presence, its size, and its longevity [5–7]. Such support can come from either of two kinds of sources: institutionalized sources like public health departments, or “mobilization from below” in the form of social movements. Institutional support from health officials, medical and public health researchers, church leaders, law enforcement, and/or school administrators can provide support and outreach for a program [4–7, 37, 39, 40, 43]. Institutional support can also come in the form of funding for public policies and programs. We can think of this as a general willingness of government to spend money on social services for the public good. Here, we hypothesize that drug treatment is generally a “liberal” response [4, 47, 48] to the problem of drug misuse, and that financial or other support from policy and other institutional sources for drug treatment programs therefore reflects a higher degree of “liberalism” than does a lack of such support. *Mobilization from below:* Social movements, ranging from the feminist health movement to AIDS activism, have helped to shape programs, and policies in the U.S. (such as treatment services, health care reform, and AIDS policy), and have also helped to shape public opinion about health-related issues (such as the de-stigmatization of groups, including PWID) [5, 6, 9, 14, 53–56]. As an example of the potential influence of social movements on programs and policies for PWID specifically, there has been support for the hypothesis that gay political influence and concern among the gay community regarding HIV/AIDS prevention (including the direct involvement of gay and lesbian activists in ACT UP) helped form and sustain harm reduction programs for PWID in the US [5–7].

*Service symbiosis* occurs when the increased or newly introduced presence of one type of service is associated with increases in other services; we have added this domain to the standard TCA model. For the present research, specifically, the presence of syringe exchange programs is hypothesized to be associated with higher levels of other drug treatment. This hypothesis is based on reports that syringe exchange programs provide many referrals to treatment [28, 47, 48, 55].

## Material and methods

### Unit of analysis and sample

The unit of analysis in this study is the MSA. The US Census Bureau and Office of Management and Budget define an MSA as a set of contiguous counties that include one or more central cities of at least 50,000 people that collectively form a single cohesive socioeconomic unit, defined by inter-county commuting patterns and socioeconomic integration [57]. The MSA was selected as the unit of analysis because data were readily available at this geographic level and because it is posited that MSAs are meaningful epidemiologic units with which to study injectors and services designated for them [3, 4]. Also, it is appropriate to include counties that comprise MSAs, as opposed to including only counties containing central cities, given that drug-related epidemics travel from central cities to their surrounding suburbs, as injectors often live in suburbs but buy drugs and perhaps receive drug-related social services in the central city [3, 4].

The sample of MSAs included in the present study was obtained by selecting all MSAs (*N* = 96) in the United States which had a population greater than 500,000 in 1993. Six of these MSAs (Gary, IN; Hartford, CT; New Haven-Bridgeport-Danbury, CT; Phoenix-Mesa, AZ; San Juan, PR; and Tucson, AZ) are missing from the present analyses because they did not report treatment data necessary to estimate treatment coverage, resulting in a sample of 90 MSAs for the present study.

Because this is a study of 90 MSAs with populations of 500,000 or more in 1993 that had data available on our key variables, our sample is a fully enumerated universe. This means there is no sampling error and that *p*-values are not meaningful as estimates of the probability of arriving at estimates based on chance introduced by selecting a sample. Nonetheless, we report statistical significance as a heuristic guide to the importance of variables in our equations. We compute them as if we had a random sample of MSAs, but report results as “pseudo-*p*-values” to guide our interpretation (as in previous articles: [19, 22]. Thus, for purpose of this paper, we used *pseudo-p < 0.05* as a heuristic criterion.

### Dependent variable: calculating drug treatment coverage

We calculated treatment coverage rates for each year from 1993 to 2007 (excluding years 1994, 1999, and 2001 due to data missingness) using information from two databases from the Substance Abuse and Mental Health Service Administration (SAMHSA) [58–61], and estimates of PWID from previous research [62]. Table 1 describes each database utilized to calculate treatment coverage rates. We define treatment coverage as the ratio of PWID in treatment to PWID in the MSA. Treatments included in our coverage estimates are residential or ambulatory inpatient/outpatient care, detoxification services and methadone maintenance therapy at publicly- and privately-funded drug treatment agencies receiving public funds. These are facilities licensed, certified, or otherwise approved by State treatment agencies to provide substance use treatment.

**Table 1:** Description of Data Sources Utilized to Calculate Drug Treatment Coverage Rates

1) Proportion of treatment entrants who indicated that they injected substances intravenously in each MSA and year (1993–2007) as reported by the Treatment Episode Data Set (TEDS) [58];	
2) Total number of drug users in drug treatment as of October 1 of each year reported by the Uniform Facility Data Set (UFDS) for 1993, 1995, 1996–1998 [59, 60] and the National Survey of Substance Abuse Treatment Services (NSSATS) for 2000, 2002–2007 [61];	
3) Total estimated number of PWID in each MSA and year (1993–2007) as calculated and reported by Tempalski and colleagues [62].	

Treatment coverage for PWID is estimated using TEDS and UFDS/N-SSATS. We use both TEDS and UFDS/N-SSATS to calculate treatment coverage to maximize validity and reliability of our estimates. Each of our data set differs in counts of drug treatment clients. TEDS counts each admission in a given year. Therefore, an individual admitted to treatment twice in a calendar year is counted as two admissions which inflates annual treatment entries, but only produces bias in the proportion of entrants who are PWID to the extent that such double-counting varies systematically by route of administration. In contrast, UFDS/N-SSATS is a one day census of treatment.

The following equation calculates drug treatment coverage rate^1^:

$$ Ajt=\left( Djt\ast \left( Bjt/ Cjt\right)\right)/ Ejt\ast 100 $$

where,

*Ajt* = treatment coverage rate for an MSA *j* in year *t*

*Bjt* = number of PWID entering drug treatment as reported by TEDS for an MSA *j* in year *t*

*Cjt* = number of PWID and number of non-injectors entering drug treatment as reported by TEDS for an MSA *j* in year *t*

*Djt* = number of drug users entering drug treatment reported by UFDS/N-SSATS for an MSA *j* in year *t*

*Ejt* = estimated number of PWID as estimated by Tempalski et al. 2013 [62] for an MSA *j* in year *t*.

First, the TEDS data series identifies the number and attributes of clients who enter substance use treatment programs that receive any state and federal funding. From TEDS, we calculated the proportion of treatment entrants who reported they injected drugs as a mode of administration. Our second SAMHSA data source comes from the annual census of drug treatment facilities originally referred to as the UFDS – but since renamed the N-SSATS. UFDS/N-SSATS data measure client characteristics and use of privately- and publicly-funded substance use treatment programs in the U.S. on October 1 for each year. However, UFDS/N-SSATS data were unavailable for 1992, 1994, 1999, and 2001. As a result of this limited availability, our coverage estimates were only created for years where data were available. Thus, our final drug treatment coverage estimates only provide data for 1993, 1995, 1996–1998, 2000, and 2002–2007.

### Calculating number of PWID

Because estimation of the total numbers of injectors is discussed in detail elsewhere [62], it is described only briefly here. Tempalski and colleagues first estimated the number of PWID in the US each year from 1992 to 2007 and then apportioned these estimates to MSAs using multiplier methods. Four different types of data indicating drug injection were used to allocate national annual totals to MSAs, creating four distinct series of estimates of the number of injectors in each MSA. These estimates rely on using (1) HIV counseling and testing data from the Centers for Disease Control (CDC) [63]; (2) SAMSHA’s UFDS and TEDS data [58–61]; (3) CDC’s diagnoses of PWIDs with HIV/AIDS [63]; and (4) an estimate derived from published estimates of the number of injectors living in each MSA in 1992 [64] and in 1998 (3). Each series was smoothed over time using loess regression and the mean value of the four component estimates was taken as the best estimate of PWID for that MSA and year. In order to avoid circularity, the estimated numbers of PWID in the population used in this study modify the Tempalski estimates [62] so that they do not rely on data on the numbers of PWID in drug treatment from SAMSHA.

### Independent variables

Data at the MSA-level were available on a range of variables measuring the theoretically supported domains of program *need, resource availability, institutional opposition, organized support, and service symbiosis*. Table 2 describes the statistical distribution of all of these independent variables across MSAs and within each theoretical domain. Additional file 1: Table S1 depicts the bivariate correlations among all variables.

**Table 2:** Statistical description of independent variables across MSAs & across all years for which outcome data was available

Variable, Outcome years to which lagged variables were matched (where 1994, 1999, and 2001 are missing)	Mean (SD)	Median (Q25- Q75)	Minimum	Maximum	Data Source
Need					
AIDS diagnosis per 10,000 population, 1997–2007*	8.88 (7.18)	6.65 (4.09–1-.40)	0.68	55.59	CDC AIDS Surveillance, 2010^1^
HIV prevalence rate among PWID, 1996–2006**	8.09 (6.55)	5.90 (3.70–10.0)	1.90	43.50	Tempalski et al., 2009^2^
Resource Availability: General resources					
Percent of population in poverty (1993–2007)***	11.55 (3.47)	10.98 (9.42–12.88)	4.34	29.20	US Census Bureau, 1990^3^
Unemployment rate (1993–2007) ***	5.35 (1.90)	5.10 (4.20–6.10)	1.80	15.90	US Census Bureau, 1990^3^
Median household income (1993–2007) ***	46,505.35 (9040.40)	44,359.76 (40,225.57–51,088.0)	29,554.99	83,318.0	US Census Bureau, 1990^3^
Long-term debt per capita, 1993–2007^a^	3.65 (1.69)	3.33 (2.38–4.59)	0.64	10.60	Surveys of Govt Finances, 2007^4^
Specialized resources					
Percent community & public health researchers & social workers in the workforce (1993–2007) ***	0.75 (0.28)	0.75 (0.62–0.90)	−0.28	1.63	Bureau of Health Professions Area Resource File, 2012^5^
Health expenditures per capita, 1993–2007^a^	0.09 (0.07)	0.07 (0.04–0.14)	0.002	0.60	Surveys of Govt Finances, 2007^4^
Institutional Opposition: Legal repressiveness/penalties					
Drug arrests rate for possession of heroin or cocaine per PWID (1994–2007) **	14.10 (11.19)	11.50 (6.18–18.82)	0.05	69.02	FBI’s Uniform Crime Reporting, 2010^6^
Correction expenditures per capita, 1993–2007^a^	0.06 (0.03)	0.05 (0.04–0.16)	0.00	0.16	Surveys of Govt Finances, 2007^4^
Racial structures (1993–2007) ***					
Ratio of Black to White median household income	0.71 (0.15)	0.70 (0.62–0.76)	0.24	1.32	Center on Comparative Urban & Regional Research (CCURR), 2000^7^
Ratio of Black to White poverty	3.37 (0.93)	3.37 (2.78–3.86)	0.63	7.15	CCURR, 2000^7^
Ratio of Black to White unemployment	2.60 (0.59)	2.57 (2.21–2.94)	0.85	5.19	CCURR, 2000^7^
Institutional Support: “Liberalism” of public policies					
Right-to-work-state, 1993^a^	Yes = 37%; No = 63%		0	1.0	Right to Work Legal Defense, 2000^8^
Education expenditures per capita, 1993–2007^a^	1.28 (0.31)	1.23 (1.06–1.45)	0.63	3.53	Surveys of Govt Finances, 2007^4^
Pressure from below					
Number of types of “early” groups per 10,000 population, 1993^a^	0.003 (0.004)	0.00 (0.00–0.005)	0.00	0.02	NAMM,1993; Brown & Beschner, 1993; NIDA, 2001^9^
Service symbiosis					
Ever had syringe exchange program, since 1993^a^	Yes = 47%; No = 53%		0	1.0	Beth Israel Medical Center; 2000^10^

* 5 year lag; ** 4 year lag; *** 3 year lag; ^a^ no lag associated with variable

^1^ Centers for Disease Control and Prevention. Request data CDC AIDS Surveillance Data, 2010. Atlanta, GA: Centers for Disease Control and Prevention

^2^ HIV Prevalence Rates as estimates by Tempalski, B., Pouget, E.R., Cleland, C.M., et al. (2013). Trends in the population prevalence of people who inject drugs in US Metropolitan Areas 1992–2007. PLoS ONE, 8 (6) e64789

^3^ US Census Bureau (1990) Housing and Household Economic Statistics Division: Poverty Index. In: Bureau UC, editor. Washington, DC

^4^ US Census of Governments. County area finances file, 1992, 1997, 2002, 2007. Washington, DC: US Census Bureau; 1992, 1997, 2002, 2007

^5^ Health Area Resources and Services Administration, Health Professional Shortage Area Website: http://www.hrsa.gov/shortage/. 2012

^6^ U.S. Department of Justice. Uniform Crime Reporting Statistics. County-level detailed arrest and offense data. 1993–2010

^7^ University at Albany, Lewis Mumford Center for Comparative Urban and Regional Research. 1990 race and residential segregation statistics. Available at: http://mumfordl.dyndns.org/cen2000/data.html. Accessed December 15, 2006

^8^ National Right to Work Legal Defense Foundation, http://www.nrtw.org/rtws.htm

^9^ Group types are: (1) chapters of National Association of Methadone Advocates by the end of 1993, or (2) presence of an “early outreach project,” defined as a participant in the NIDA-funded NADR or Cooperative Agreement projects

^10^ Beth Israel National Survey of Syringe Exchange Programs [database]. New York, NY: Beth Israel Medical Center; 1993–2007

## Analytic approach

We utilize a series of mixed-effects models [65] to examine trends in drug treatment coverage across the study period and to test all study hypotheses. This method used maximum likelihood estimation to assess the associations of interest while adjusting for variance shared within MSAs across time.

### Lag

Where possible, we included time lags in our measurement strategy to ensure that our independent variables had time to affect treatment coverage. We measured treatment coverage for 1993–2007. Thus, we measured each independent variable before 1993. We chose three-year lags in most cases (e.g., demographic and economic variables collected in the 1990 US Census). Our inclusion of time lags also reflects the likelihood that many of these variables change slowly (and that therefore any change in the outcome which might occur as a response to or in accordance with changes in other setting characteristics could take years), and acknowledges the time required to create or change treatment programs. There are also three variables (presence of SSPs; Right-to-work State; and Number of types of “early” groups) which were only measured once, in 1993, and for which change over time was not assessed. To facilitate interpretation of intercepts and of the effects of predictors which interact with time, we centered independent variables at the first year for which we measured them.

### Mixed-effects models

Growth curve models were utilized first in order to assess the nature of the relationship between time and treatment coverage. Linear, quadratic, and cubic functions for time were modeled in this “univariate” first step in order to assess the functional form of change in treatment coverage over time. Next, in order to select the most empirically relevant set of independent predictors of treatment coverage from the large number (relative to the number of MSAs) of theoretically-relevant potential independent variables, we developed a four-step process. Each stage in this four-step process utilized a logarithm-transformed version of the dependent variable to address the non-normal distribution of the treatment coverage variable.

#### Step 1: bivariate model section

First, we conducted bivariate analyses to determine which independent variables might be associated with treatment coverage. For each of the potential independent variables, separately, we used mixed-effects models [66] to assess the strength and nature of its relationship to treatment coverage. We developed three models appropriate for understanding potentially nuanced relationships varying in a curvilinear manner over time in a multilevel framework:

$$ \mathrm{Model}\kern0.28em 1\Big)\kern0.28em \hat{Y}=A+B+C\kern0.28em Time+ Tim{e}^2 $$

$$ \mathrm{Model}\;2\Big)\;\hat{Y}=A+B+C+ Tim e+ Tim{e}^2+B\ast Tim e+B\ast Tim{e}^2 $$

and

$$ \mathrm{Model}\;3\Big)\;\hat{Y}=A+B+C+ Tim e+ Tim{e}^2+C\ast Tim e+C\ast Tim{e}^2 $$

A = Intercept

Ŷ = predicted treatment coverage

B = baseline values of each potential independent variable

C = change in independent variable baseline at each stage.

We then compared each of these three nested models for each potential independent variable, and selected the model with the “best fit” for each construct based on *Akaike’s Information Criterion* (AIC) [67, 68]. The “best model” was chosen based on the following criterion: Model 1 was the default “best” model, unless the AIC for Model 2 or Model 3 was lower than that for Model 1 by at least 2, in which case the model with the lowest AIC was chosen. This criterion was utilized as a mechanism for ensuring that interactions were only included if they improved model fit by a meaningful margin.

#### Step 2: bivariate analyses

In the second step, we ran the “best fit” model for each construct using standardized variables (z-scores), and compared standardized coefficients from these “best fit” models for each potential independent variable to determine eligibility for entry into the next step of analysis (domain analysis). The somewhat standard use of *pseudo-p*-values as a criterion for bivariate selection of independent variables into multivariate models was not possible due to the nature of the “best fit” mixed models, which included multiple parameters that together contributed to the ability of each construct as a whole to meaningfully explain variation in treatment coverage. Standardized coefficients, however, are an appropriate measure of effect size [69] which can serve as indicators of the relative explanatory importance of each variable in predicting treatment coverage. Given our desire to consider, as a whole, the ability of the multiple parameters composing each construct to predict our outcome, we summed the standardized coefficients from the multiple parameters composing each construct. Considering Ferguson’s [69] recommendation of a 0.2 minimum effect size for “strength of association” measures, including standardized coefficients, we used the criterion that the sum of the absolute value of the standardized coefficients from the best fit model for the construct (not including the coefficients for the Intercept or for Time or Time^2^, but including the coefficients for B, for C, and for any applicable interactions) must be equal to or greater than 0.25. A lower criterion of 0.20 was applied to constructs for which only one or two coefficients were included in its “best fit” model, either due to Model 1 (with no interactions) being selected, or due to the construct being time-invariant and therefore not having change scores included in its model. Because standardized versions of all variables were used in these analyses, the comparison of their standardized coefficients from models predicting treatment coverage should serve as a reasonable comparison of their relative explanatory importance in understanding variation in treatment coverage.

#### Step 3: domain analyses

In the third step, we selected only independent variables which met the criterion applied to the bivariate analyses in Step 2, and ran a set of mixed-effect models, each of which included the “best fit” models for each of the eligible constructs in a specific theoretical domain. The same criteria used in Step 2 for bivariate analyses (>.25 or > .20 sum of model coefficients) were then applied to the results of these domain analyses to determine eligibility for inclusion of each construct in the final analytic model. This step allowed us to limit potential multicollinearity by identifying the most empirically important predictors of treatment coverage from a set of highly conceptually related variables and eliminating the rest.

#### Step 4: multi-domain analyses

In the next step, the “best fit” models from all domains which met the eligibility criterion in Step 3 were included into a single mixed-effects multi-domain model predicting treatment coverage, to estimate the relationships of each eligible independent variable to treatment coverage, net of the predictive influence of all other eligible independent variables. Finally, to achieve the most parsimonious multi-domain model, model parameters which did not meaningfully contribute to the multi-domain prediction of treatment coverage were identified for removal using the following process: model AIC was compared among versions of the multi-domain model which systematically and individually removed either a) interactions with Time^2^; b) non-“significant” interactions; or c) constructs for which neither the baseline nor change score were “significant” predictors of treatment coverage. The model with the fewest parameters and lowest AIC was selected. Constructs with a “significant” coefficient at either their baseline or change score were not removed from the original multi-domain model, and no parameters were removed for which removal resulted in a > 2.0 increase in AIC. This process allowed us to assess the importance to the model of including each interaction and each non- “significant” construct. All analyses were conducted using SAS software. Mixed-effects models were conducted using PROC MIXED in SAS [66].

## Results

### Descriptive statistics

A comparison of the beginning and end points of the study period reveals little variation in treatment coverage over time. Coverage overall was very similar in 1993 and 2007 (Additional file 1: Table S2). Mean coverage was only 6.4% (SD = 4.5) in 2007, which was quite similar to the estimated 6.7% coverage in 1993 (SD = 3.7). Median treatment coverage was also quite similar in 1993 (5.6%) and in 2007 (5.2%) among injection drug users in 90 US MSAs.

### Growth curve model

**Fig. 2:**
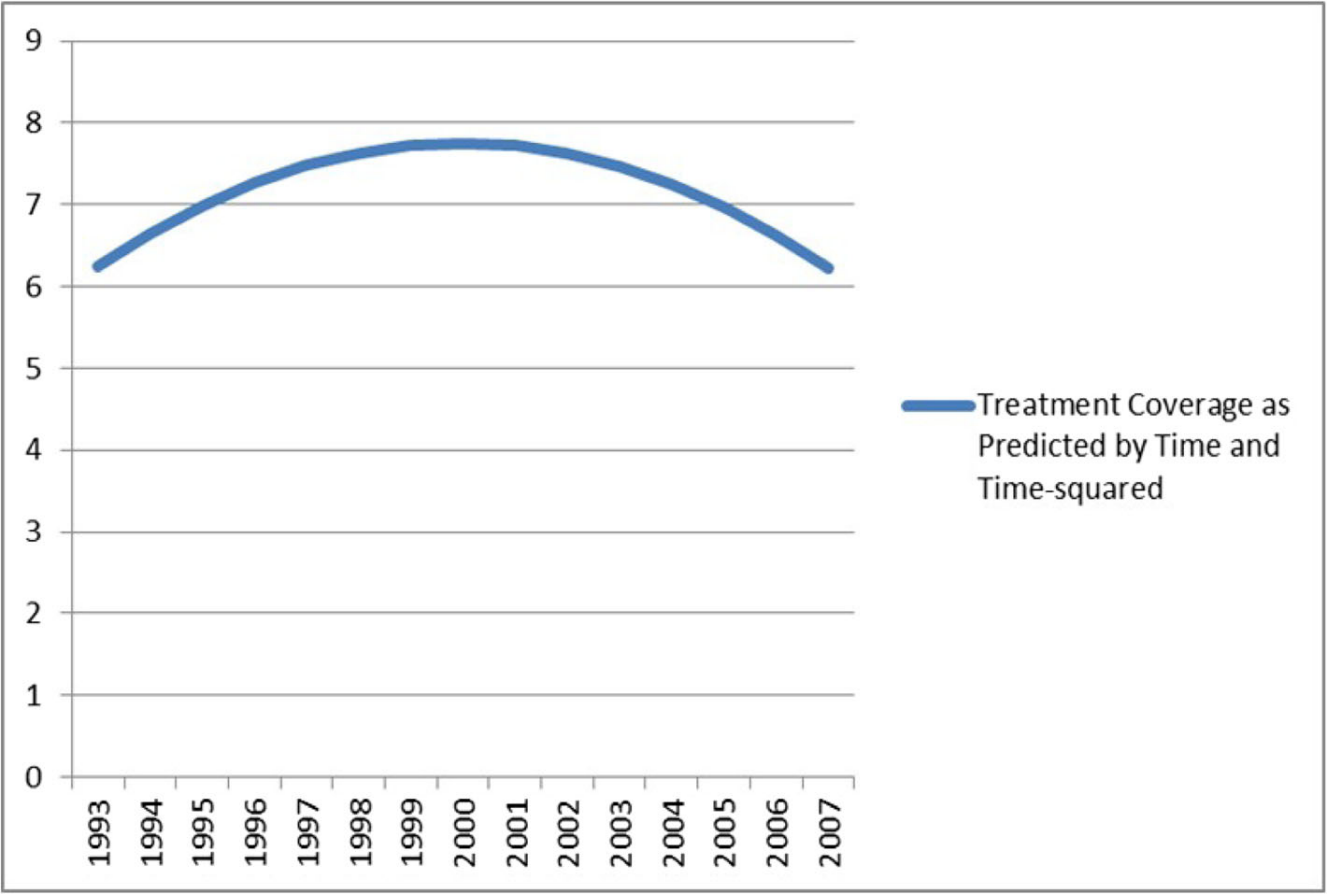
Quadratic Growth Curve for Treatment Coverage, 1993–2007

Mixed-effects growth curve models were utilized to examine the nature of average changes in treatment coverage between the 1993 and 2007 time points (linear, quadratic, and cubic). The model for a quadratic function of time contained “significant” coefficients for both linear time (β = 0.43; S.E. =0.10; *pseudo-p* < 0.0001) and quadratic time (β = − 0.03; S.E. = 0.01; *pseudo-p* < 0.0001), suggesting that there is a “significant” curvilinear trend for change in treatment coverage over time, averaging across MSAs. When modeled as a quadratic function of time (see Fig. 2), treatment coverage increases across MSAs, on average, from 1993 to 2000, and then decreases across MSAs, on average, from 2001 to 2007. The curvilinear increase in treatment coverage rising in the 90s and peaking in 2000 might have been due to the rise in nationwide opiate overdoses which may have driven some increase in treatment availability during this period [70, 71].

### Bivariate and multivariate models

Table 3 shows the results of both the bivariate analyses and the domain**-**specific analyses. A logarithm transformation was used on the dependent variable for these and all subsequent models to address the non-normal distribution of the treatment coverage variable. Ten out of seventeen potential constructs met the eligibility criterion based on bivariate analysis to be included in the domain specific analysis. Of the ten constructs included in the domain analyses, nine met the criterion to be included in the multi- domain model.

**Table 3:** Standardized Predictors in Bivariate and Domain-Specific Analyses

Domain	Variable	Step 2: Bivariate^1^ Analyses - β (SE)	Step 3: Domain Analyses - β (SE)
Need	Intercept	− 0.288 (0.168)	
	AIDS per 10 k population	− 0.056 (0.097)	
	Change AIDS among PWID per capita	0.062 (0.067)	
	Intercept	− 0.617 (0.153)	− 0.617 (0.153)
	HIV among PWID per capita	− 0.193 (0.128) ^†^	− 0.193 (0.128) ^†^
	Change HIV among PWID per capita	0.066 (0.058) ^†^	0.066 (0.058) ^†^
	HIV among PWID per capita * Time	0.051 (0.027) ^†^	0.051 (0.027) ^†^
	HIV among PWID per capita * Time^2^	− 0.001 (0.002) ^†^	− 0.001 (0.002) ^†^
Resource Availability: General Resources	Intercept	− 0.054 (0.092)	− 0.263 (0.211)
	Percent of population in poverty	− 0.185 (0.084) ^†^	− 0.224 (0.122) ^†^
	Change percent of population in poverty	0.355 (0.163) ^†^	0.398 (0.172) ^†^
	Change percent poverty * Time	− 0.068 (0.028) ^†^	− 0.067 (0.029) ^†^
	Change percent poverty * Time^2^	0.003 (0.001) ^†^	0.003 (0.001) ^†^
	Intercept	− 0.266 (0.124)	
	Long-term debt per capita	− 0.168 (0.089)	
	Change long-term debt per capita	0.015 (0.054)	
	Intercept	− 0.179 (0.103)	− 0.263 (0.211)
	Unemployment rate	0.078 (0.087) ^†^	0.221 (0.096) ^†^
	Change unemployment rate	0.251 (0.068) ^†^	0.217 (0.076) ^†^
	Change unemployment rate * Time	− 0.093 (0.017) ^†^	− 0.093 (0.019) ^†^
	Change unemployment rate * Time^2^	0.005 (0.001) ^†^	0.005 (0.001) ^†^
	Intercept	− 0.545 (0.196)	− 0.263 (0.211)
	Median household income	0.217 (0.084) ^†^	0.109 (0.108) ^†^
	Change median household income	− 0.535 (0.192) ^†^	− 0.141 (0.211) ^†^
	Change household income * Time	0.070 (0.026) ^†^	0.008 (0.030) ^†^
	Change household income * Time^2^	− 0.003 (0.001) ^†^	0.000 (0.001) ^†^
Resource Availability: Specific Resources	Intercept	− 0.078 (0.086)	− 0.335 (0.117)
	Community/public health research & social work workforce	0.445 (0.073) ^†^	0.462 (0.075) ^†^
	Change in public health research & social work workforce	− 0.001 (0.051) ^†^	0.001 (0.053) ^†^
	Intercept	− 0.318 (0.122)	− 0.335 (0.117)
	Health expenditures per capita	0.229 (0.092) ^†^	0.154 (0.080) ^†^
	Change health expenditures per capita	− 0.042 (0.053) ^†^	− 0.061 (0.051) ^†^
Institutional Opposition: Legal Repressiveness	Intercept	− 0.073 (0.092)	
	Drug arrests rate for possession of heroin or cocaine	0.141 (0.085)	
	Change drug arrests rate for possession of heroin or cocaine	0.005 (0.026)	
	Intercept	− 0.353 (0.120)	
	Correction expenditures per capita	− 0.031 (0.092)	
	Change correction expenditures per capita	− 0.088 (0.040)	
Institutional Opposition: Racial Structure	Intercept	− 0.070 (0.094)	
	Ratio of Black to White median household income	0.040 (0.088)	
	Change Ratio of Black to White median household income	0.042 (0.050)	
	Intercept	− 0.271 (0.106)	− 0.271 (0.106)
	Ratio of Black to White Poverty	− 0.103 (0.081) ^†^	− 0.103 (0.081) ^†^
	Change Ratio of Black to White Poverty	0.763 (0.202) ^†^	0.763 (0.202) ^†^
	Change Ratio of Black to White Poverty * Time	− 0.077 (0.032) ^†^	− 0.077 (0.032) ^†^
	Change Ratio of Black to White Poverty * Time^2^	0.002 (0.001) ^†^	0.002 (0.001) ^†^
	Intercept	− 0.067 (0.093)	
	Ratio of Black to White unemployment	− 0.074 (0.087)	
	Change Ratio of Black to White unemployment	0.100 (0.036)	
Institutional Support: “Liberalism” of Public Policies	Intercept	− 0.078 (0.079)	− 0.291 (0.125)
	Right-to-work-state (in 1993)	− 0.459 (0.071) ^†^	− 0.463 (0.082) ^†^
	Intercept	− 0.276 (0.133)	− 0.291 (0.125)
	Education expenditures per capita	0.222 (0.088) ^†^	0.034 (0.083)
	Change education expenditures per capita	0.002 (0.041) ^†^	− 0.007 (0.041)
Institutional Support: Group Pressure	Intercept	− 0.078 (0.094)	
	Number of “early” groups (in 1993) per 10 k population	− 0.036 (0.086)	
	Intercept	− 0.078 (0.089)	− 0.078 (0.089)
Institutional Support: Service Symbiosis	Ever had syringe exchange program, by 1993	0.230 (0.082) ^†^	0.230 (0.082) ^†^

^1^ Each “bivariate” model and each model in the domain analyses also includes coefficients for both Time and Time.^2^ These coefficients are not listed due to space restrictions, and, like the coefficients for the Intercepts, do not contribute to the calculation of coefficient sums

† Covariates with this symbol contributed to a sum that met the criteria for inclusion into the next analytic step.

Additional file 1: Table S3 shows the results of the first multi-domain model which includes all qualifying independent variables from across the domain analyses. Table 4 shows the results of the final multi-domain model, from which parameters were removed based on

**Table 4:** Results from Parsimonious Multi-Domain Mixed-Effects Model

Independent Variable	β	SE	*Pseudo-p*
Intercept	−0.806	0.169	< 0.0001
Time (Years since Baseline)	0.169	0.041	< 0.0001
Time^2^ (Years-since-Baseline, squared)	−0.008	0.002	0.001
Need			
Baseline HIV Prevalence among PWID per capita	−0.360	0.097	0.0003
Change in HIV among PWID per capita	0.088	0.057	0.123
Baseline HIV among PWID per capita * Time	0.039	0.012	0.001
Resource Availability: General resources			
Baseline Percent of Population in Poverty	−0.486	0.081	< 0.0001
Change in Percent of Population in Poverty	−0.040	0.044	0.357
Baseline Unemployment Rate	0.312	0.081	0.0002
Change in Unemployment Rate	0.407	0.127	0.002
Change in Unemployment Rate * Time	−0.130	0.031	< 0.0001
Change in Unemployment Rate * Time^2^	0.008	0.002	< 0.0001
Resource Availability: Specific resources			
Baseline Public Health and Social Work Workforce	0.425	0.071	< 0.0001
Change in Public Health and Social Work Workforce	−0.054	0.054	0.321
Racial structures			
Baseline Ratio of Black to White Poverty	−0.138	0.072	0.058
Change in Ratio of Black to White Poverty	1.269	0.307	< 0.0001
Change in Ratio of Black to White Poverty * Time	−0.171	0.052	0.001
Change in Ratio of Black to White Poverty * Time^2^	0.006	0.002	0.009

the process of AIC comparisons described above. Findings from the final model in Table 4 are described below, followed by a brief comparison of coefficients from the two multi- domain models. In the final model both Time (β = 0.169; *pseudo-p* < 0.0001) and Time^2^ (β = − 0.008; *pseudo-p* = 0.001) were found to be statistically significant predictors of treatment coverage, net of the effects of all other independent variables in the model.

From the *need* domain, HIV prevalence among PWID was included in the final model. Higher HIV prevalence among PWID at baseline was found to predict significantly lower treatment coverage (β = − 0.360; *pseudo-p* = 0.0003), on average. The interaction of HIV at baseline with Time was also statistically significant (β = 0.039; *pseudo-p* = 0.001), indicating that baseline values of HIV significantly predict variation in the trajectory of treatment coverage over time.

From the resource availability domain, percent of population in poverty; unemployment rate; and community, public health, and social work workforce were included in the final model. Both higher unemployment rates at baseline (β = 0.312; *pseudo-p* = 0.0002) and increases from baseline in unemployment (β = 0.407; *pseudo-p =* 0.002) were found to predict significantly higher treatment coverage, on average. In addition to a significant interaction with Time, the interaction between change in unemployment and Time^2^ was statistically significant (β = 0.008; *pseudo-p* < 0.0001), indicating that change in unemployment rate from baseline significantly predicted variation in treatment coverage trajectories over time.

Although change in poverty rate from baseline was not found to be a significant predictor of treatment coverage (β = − 0.040; *pseudo-p* = 0.357), higher poverty rate at baseline was found to predict significantly lower levels of treatment coverage, on average (β = − 0.486; *pseudo-p* < 0.0001). Larger community, public health, and social work workforces at baseline were also found to predict significantly higher treatment coverage levels, on average (β = 0.425; *pseudo-p* < 0.0001).

From the institutional opposition domain, only the black/white poverty disparity construct was included in the final model. Although the level of disparity in poverty rates among Black and White households at baseline was not significantly predictive of variation in treatment coverage (β = − 0.138; *pseudo-p* = 0.058), on average, increases in black/white poverty disparity from baseline predicted significantly higher treatment coverage among MSAs, on average (β = 1.269; *pseudo-p* < 0.0001). In addition to its interaction with Time, the interaction of change in Black/White poverty disparities from baseline with Time^2^ was also statistically significant (β = 0.006; *pseudo-p* = 0.009), suggesting that change in Black/White poverty disparities significantly predicted variation in treatment coverage trajectories over time. None of the variables from either the institutional support or the service symbiosis domains met all criteria for inclusion in the final model.

A comparison of this final model to the models in S3 (which is the less parsimonious model including all variables that were originally eligible for multi-domain analysis based on the results of domain analyses) illustrates that the positive or negative valence of the coefficients for all independent variables except one (the non-significant coefficient for change in percent population in poverty) remained constant across these two models (and also across all tested versions of the multi-domain model), suggesting stability of our findings across models with various non-significant parameters (and other parameters not contributing to overall model fit) removed.

## Discussion

As previously reported by Tempalski and colleagues [1–4], treatment coverage for PWID in large US metropolitan areas is far below international standards. Some European Union countries, for example, maintain coverage levels of 65% or higher [72]. Although the overall level of treatment coverage for PWID in our 90 MSAs was similarly low at the beginning (6.7%), and end (6.4%) of the study period, it changed significantly during this time period, increasing on average until 2000, then decreasing to its original level.

Contrary to our hypothesis based on the TCA, epidemiologic need, as measured both by the prevalence of AIDS cases per 10,000 population and by HIV prevalence among PWID, seems not to be associated with change in treatment coverage for PWID over time. The present study does find that baseline need as measured by 1993 HIV prevalence rate predicted both level of treatment coverage and variation in trajectories of treatment coverage. These findings, that epidemiologic changes in HIV after 1993 did not correspond with related changes in treatment coverage therefore suggest that service systems are not adequately or efficiently attending to changes in need. They could suggest that *need* is not, in fact, engendering a direct service provision response at all, which would be consistent with the findings of our previous studies [4–7].

Such results have important public health implications given the current opiate overdose epidemic, and may indicate that the US should find new ways to allocate resources to drug treatment programs in order to allow responses to a changing need environment. Clearly, drug treatment programs are the basic tools with which public health agencies try to influence and reduce the harms associated with substance misuse. They are thus central to our efforts in helping those vulnerable to substance misuse lead healthy lives. Yet, the US today still struggles with implementing some of the most basic of services for those in need. Public policies need to address the broad individual, environmental, and societal factors that influence substance misuse and its consequences. For example, local politicians have power to shape the nature of care and funding for publicly-funded treatment programs. State licensing and financing policies can provide incentives to programs to offer the full continuum of care (i.e., residential, outpatient, continuing care, and recovery supports) including behavioral treatments and therapy for mental issues and opiate medications, such as buprenorphine. Service providers, harm reduction advocates and researchers can also work toward developing local levels of social and policy support for expanding continuum of care programs in areas experiencing program NIMBYism [39, 40, 43, 46].

As hypothesized, results imply that resource availability does seem to shape service provision of drug treatment. Our model indicates that general resources (both poverty and unemployment rate) are important economic indicators that affect drug treatment coverage (either directly or indirectly). Here, consistent with hypotheses, higher poverty at baseline was found to predict lower levels of treatment coverage.

However, higher baseline rates of unemployment and increases in unemployment over time were associated with higher levels of treatment coverage, and change in unemployment also significantly predicted variation in treatment coverage trajectories over time. This relationship between unemployment and treatment coverage would therefore appear to be driven by processes which are not accounted for by the TCA. One plausible explanation for this positive relationship between unemployment and treatment coverage may be that higher levels of unemployment result in more people entering treatment due to job placement programs that require treatment, or simply due to the availability of people who are not working to participate in treatment [73–77]. For example, research by Popovici and French (2013) [78] and Henkel (2011) [77] have found that both drinking and smoking patterns increase when the economy declines and unemployment rate increases. Both research suggests that the need for treatment services appear to be procyclical with economic turndown. As such, our finding suggests the need for more research and understanding on the effect of changes in unemployment and drug treatment coverage over time.

Additionally, we hypothesized that specialized resources might suggest a concentration of local assets that directly supports a narrow range of programs and/or support a movement for a program or expansion of a program. Previous research measured specialized resources useful for the community action being studied. Such actions have included SPP presence and HIV testing and treatment among PWID [4–7, 38–40], as well as a broad range of programs such as providing condom distribution interventions or programs [20, 21, 36], or specialized programs for treatment of people who are mentally ill [40, 41]. In the present study we found that, consistent with our hypotheses, having a larger community, public health, and social work workforce in MSAs predicted higher treatment coverage levels.

As such these findings strengthens the argument that specialized resources in support of treatment provision are needed especially in communities hit hard by the opioid epidemic. Assets that directly supports a narrow range of programs (i.e., opioid overdose prevention, naloxone distribution) and implementation strategies (i.e., coordinated multi-system & multi-sector public health response driven by community engagement) can take into account special needs and resources in local communities. More specifically, service organizations should articulate the aims of the proposed service in terms that fit in with the local community’s epidemiological needs. Thus, increasing specialized resources for drug treatment may be an issue of pushing for more local government funding for treatment services and developing local coalitions such to do so.

Variables within the institutional opposition domain, such as drug arrests and correction expenditures (i.e., organized and potential opposition to programs), which were hypothesized to predict declines in or lower levels of services, were not found to be associated with treatment coverage. However, evidence from our model suggests that racial structures within this domain (ratio of Black to White poverty) do significantly predict treatment coverage for PWID. Here, we found that in MSAs where Black to White poverty disparity has increased over time, treatment coverage on average is higher than it is in MSAs which experienced a decrease in poverty disparity, with change in disparity not only predicting level of treatment coverage, but also change in treatment coverage over time.

One possible explanation for this relationship could be that, as economic disparities between racial groups increase, and relative disadvantage increases among already disadvantaged groups, drug use may also increase, resulting in a higher number of individuals entering drug treatment [79, 80]. This finding may therefore have important implications both for future directions in epidemiological research which aims to understand factors predicting need, as well as for service systems which strive to meet changing demands in need [81].

Historically, these data report on outcomes from the early 1990s until 2007. This was a time of high need for effective programs related to hard drug use and injection. HIV prevalence and mortality among PWUD in 1993 was at an all time high, and remained so throughout most of that decade. Overdose deaths were continuing in an exponential growth curve dating back at least until 1980 [70, 71, 82]. By standard economic indicators, this was a period of relative prosperity; the Great Recession began only near the end of 2007. Politically, this was a period of War on Drugs but also a period when drug treatment was widely supported as an HIV prevention strategy and as a way to prevent overdoses. Further, when SSPs were proposed and/or established in various cities of the US, opponents often rallied around the watchword that treatment was what was needed and that syringe exchange was a diversion of resources from treatment [43, 44, 47, 48, 82–84] Even still, SSPs are banned in 15 states (see https://www.vox.com/science-and-health/2018/6/22/17493030/needle-exchanges-ban-state-map

On the one hand, despite the claims of critics of SSPs (i.e, public injecting, inappropriate disposal of used syringes, mortality of drug use and associated illegal activity) [43, 84–88], the presence of a syringe exchange was not associated with decreases in treatment coverage. Need had only a weak relationship with treatment coverage, and changes in need were not associated with changes in coverage. Resource availability indicators (baseline poverty rate and social work workforce) were associated with treatment coverage at baseline in directions suggesting that resource lack retards treatment coverage—yet, in spite of this, at a time of relative economic prosperity, overall treatment coverage in the US did not increase.

The US is today faced with a severe crisis of overdose mortality based primarily on opioid use. Opioid use, unlike most other forms of drug use, has reasonably effective forms of medically assisted treatment available. Federal efforts like the HEALing Community initiative (see https://heal.nih.gov/research/research-to-practice/healing-communities) have been funded to conduct research into how to provide more treatment for drug users—a critical issue well-deserving of research. Nonetheless, it should be noted that HEALing Community is a research project, and is only funded and design to cover approximately 2 % of US counties.

It is unclear whether funders and policy makers will respond to the results of HEALing Communities with the needed expansion and continuum of care of drug treatment and other services for people who use drugs. Our results in this paper, and in previous research by this team [1–7, 38, 43, 44] suggest that efforts to increase treatment to have public health scale impacts on overdose deaths face severe obstacles.

## Limitations

Certain data limitations must be taken into account when interpreting the findings from the present study. As discussed in the Methods section, the limitations of both the TEDS and the UFDS/N SSATS data sources used to calculate our estimates of treatment coverage may have resulted in some bias in our estimation of the outcome variable. In addition, change in our estimated numbers of PWID in treatment in an MSA might in part result from measurement error, specifically from change in which and how many treatment facilities in an MSA respond to SAMSHA surveys. The survey response rate increased from 87% in 1995 to 94.5% in 2007, producing a 7.5% increase in reported US client totals from 1995 to 2007. SAMSHA attempts to obtain responses from all known treatment facilities, but the survey is voluntary and no adjustments for facility non- response are made. As a result, the estimated changes in treatment coverage may partially reflect changes in SAMSHA survey methodology over time. UFDS/N-SSATS data were unavailable for 1994, 1999, and 2001. Consequently, our drug treatment estimates were only created for years where data were available. Thus, our final coverage estimates only include data for 1993, 1995, 1996–1998, 2000, and 2002–2007. Finally, PWID estimates beyond 2007 were not available for our coverage estimates. As such, our data and analyses do not extend beyond 2007 due to the lack of PWID population denominators. Although exact estimates are hard to come by, estimating the contribution of drug treatment availability in preventing opioid-related morbidity and mortality among PWID is key for public health and common sense health policy in reducing harms.

## Conclusions

Programs are the basic tools with which public health agencies try to influence HIV transmission and disease progression. Despite this, however, relatively little is known about what determines their presence and reach. Much health policy discourse assumes that need for a program is associated with program presence or magnitude— however our previous studies of the determinants of drug treatment coverage and syringe exchange presence have found that need is not a predictor of these programs for PWID [4–7, 38]. The present study, similarly, has found that several key indicators of need were not related to 1993–2007 drug treatment coverage. While one 1993 indicator of need (HIV prevalence) was found by the present study to be related to differences in coverage, lack of a relationship between treatment coverage and change in need over time may still point to a lack of synergy between local need and service provision.

The present study also has found that several indicators of resource availability and economic conditions at the MSA level were related to variation in levels of treatment coverage from 1993 to 2007. These associations are especially important findings of the present study given the flux in economic conditions around the US and increased disparity among racial/ethnic groups. Specifically, a time of increased economic difficulty and instability since the early 2000’s has led to higher rates of unemployment, poverty, and evictions and foreclosures. This may have resulted in an increase in the level of economic disadvantage within neighborhoods.

Economic disadvantage has been associated with a variety of social problems, including income inequality, housing instability and crime, and to increased substance use prevalence rates [79–81, 89]. Economic changes appear to be important factors in predicting changes in treatment services [76, 90–94]. Given these empirical and theoretical links between treatment services and economic conditions, future research should consider the implications of the past and present financial conditions for treatment coverage.

## Supplementary information

**Additional file 1: Table S1.** Bivariate correlation matrix among all variables. **Table S2.** Estimated drug treatment coverage rates. **Table S3.** Results from Full Multi-Domain Mixed-Effects Model by Domain.

## Data Availability

The datasets used and/or analyzed during the current study are available from the corresponding author on reasonable request.

## Abbreviations

ACT UP: AIDS Coalition to Unleash Power
AIDS: Acquired immune deficiency syndrome
AL: Alabama
ART: Antiretroviral therapy
CA: California
CDC: Centers for Disease Control
HCV: Hepatitis C virus
HIV: Human immunodeficiency virus
KY-IN: Kentucky-Indiana
MI: Michigan
MSAs: US metropolitan statistical areas
MSM: Men who have sex with Men
NC: North Carolina
NJ: New Jersey
N-SSATS: National Survey of Substance Abuse Treatment Services
NY: New York
OH: Ohio
PA: Pennsylvania
PWID: People who inject drugs
PWUD: People who use drugs
SAMHSA: Substance Abuse and Mental Health Service Administration
SSPs: Syringe Service Programs
TCA: Theory of Community Action
TEDS: Treatment Episode Data Set
UFDS: Uniform Facility Data Set
US: United States
VA: Virginia

## Footnotes

1 During our study period, SAMSHA eliminated questions from UFDS about the number of PWIDs in a treatment program. To correct for this, we multiplied the proportion of drug users who inject drugs (from TEDS) in each MSA and year by the total number of drug users in treatment as reported by UFDS/N- SSATS. Second, these data sets differ in what they count: TEDS counts each admission in a given year, so an individual who enters drug treatment twice or more in a year is counted as two or more independent cases. In contrast, UFDS/N-SSATS is a point-prevalence survey of treatment facilities and clients on a specific date. Thus, if PWIDs differ from non-PWIDs in the ratio of admissions to those remaining in treatment, our estimates will be biased. In addition, these data do not capture medication-assisted treatment (MAT) operating out of private medical offices. Lastly, we need to acknowledge much of any system response to the opioid epidemic would have taken place after 2007, and so our data would miss those more recent changes.
