# Supplementary material for: Predictors of historical change in drug treatment coverage among people who inject drugs in 90 large metropolitan areas in the USA, 1993–2007
Source: Subst Abuse Treat Prev Policy. 2020 Jan 9;15:3. doi: 10.1186/s13011-019-0235-0 (PMC6953254; doi:10.1186/s13011-019-0235-0)
Supplement: Supplementary file 1 — Additional file 1: Table S1. Bivariate correlation matrix among all variables. Table S2. Estimated drug treatment coverage rates. Table S3. Results from Full Multi-Domain Mixed-Effects Model by Domain. [file 13011_2019_235_MOESM1_ESM.docx]

**Supplementary Table S1**

| **S1. Correlation Matrix** | 1. | 2. | 3. | 4. | 5. | 6. | 7. | 8. | 9. | 10. | 11. | 12. | 13. | 14. | 15. | 16. | 17. |
| --- | --- | --- | --- | --- | --- | --- | --- | --- | --- | --- | --- | --- | --- | --- | --- | --- | --- |
| 1. AIDS per 10k population | 1 |  |  |  |  |  |  |  |  |  |  |  |  |  |  |  |  |
| 2. HIV per capita among PWID | .490 | 1 |  |  |  |  |  |  |  |  |  |  |  |  |  |  |  |
| 3. % population in poverty | .137 | -.049 | 1 |  |  |  |  |  |  |  |  |  |  |  |  |  |  |
| 4. Long-term debt per capita | .312 | .017 | .005 | 1 |  |  |  |  |  |  |  |  |  |  |  |  |  |
| 5. Unemployment rate | .046 | .021 | .530 | -.151 | 1 |  |  |  |  |  |  |  |  |  |  |  |  |
| 6. Median house-hold income | .262 | .154 | -.573 | .166 | -.235 | 1 |  |  |  |  |  |  |  |  |  |  |  |
| 7. Community & public health workforce | .206 | .260 | -.069 | -.035 | .041 | .276 | 1 |  |  |  |  |  |  |  |  |  |  |
| 8. Health Expenditures | .013 | -.214 | -.046 | .103 | .127 | .160 | .169 | 1 |  |  |  |  |  |  |  |  |  |
| 9. Drug arrests per 10k population | .387 | .267 | .278 | -.111 | .383 | .132 | .181 | .171 | 1 |  |  |  |  |  |  |  |  |
| 10. Correction Expenditures | .347 | .123 | .100 | .295 | .157 | .195 | -.007 | .331 | .325 | 1 |  |  |  |  |  |  |  |
| 11. Black/white med income ratio | -.091 | -.151 | -.042 | -.015 | .024 | .195 | -.054 | -.029 | -.034 | .052 | 1 |  |  |  |  |  |  |
| 12. Black/white poverty ratio | -.153 | .106 | -.332 | -.113 | -.210 | .033 | .075 | .072 | -.195 | -.162 | -.220 | 1 |  |  |  |  |  |
| 13. Black/white unemployment ratio | -.026 | .095 | -.117 | .044 | -.161 | -.037 | .015 | .108 | -.145 | -.105 | -.245 | .804 | 1 |  |  |  |  |
| 14. Right-to-work State (1993) | .059 | .002 | .233 | .259 | -.185 | -.389 | -.486 | -.405 | -.117 | -.109 | -.149 | -.097 | -.070 | 1 |  |  |  |
| 15. Education Expenditures | -.007 | .095 | -.085 | .049 | -.026 | .290 | .316 | .021 | .008 | .204 | .049 | .147 | .000 | -.307 | 1 |  |  |
| 16. Early pressure groups per 10k | .209 | .002 | .083 | .069 | .074 | .084 | .141 | .142 | .038 | .054 | .058 | -.144 | -.113 | -.062 | -.087 | 1 |  |
| 17. Ever had SEP (by 1993) | .191 | .046 | -.006 | .081 | .016 | .223 | .341 | .162 | .131 | .137 | -.047 | .018 | .115 | -.296 | .028 | .060 | 1 |

1

**Supplementary Table S2**

**S2. Estimated drug treatment coverage rates**

|  | **Mean** | **SD** | **Median** | **Q25** | **Q75** | **Min** | **Max** |
| --- | --- | --- | --- | --- | --- | --- | --- |
| **Treatment Coverage*** |  |  |  |  |  |  |  |
| **1993** | 6.75 | 3.70 | 5.60 | 4.20 | 9.40 | 0.80 | 16.40 |
| **1995** | 6.71 | 4.16 | 5.65 | 3.60 | 9.70 | 0.90 | 20.60 |
| **1996** | 6.66 | 4.01 | 5.05 | 3.40 | 9.70 | 0.90 | 18.10 |
| **1997** | 6.87 | 5.47 | 5.30 | 3.00 | 9.70 | 1.10 | 28.90 |
| **1998** | 8.22 | 6.42 | 5.90 | 3.40 | 12.00 | 0.60 | 36.00 |
| **2000** | 7.87 | 5.81 | 6.05 | 3.40 | 10.30 | 0.50 | 31.80 |
| **2002** | 8.70 | 5.38 | 7.40 | 4.50 | 10.90 | 1.00 | 26.80 |
| **2003** | 7.31 | 5.36 | 5.70 | 3.20 | 9.20 | 0.90 | 27.30 |
| **2004** | 6.67 | 5.15 | 5.30 | 3.20 | 8.10 | 0.90 | 29.40 |
| **2005** | 6.50 | 4.99 | 4.85 | 2.80 | 8.30 | 1.00 | 22.70 |
| **2006** | 6.48 | 4.48 | 5.10 | 2.90 | 8.20 | 0.90 | 22.30 |
| **2007** | 6.40 | 4.51 | 5.15 | 3.20 | 8.90 | 0.90 | 19.70 |

*Proportion in treatment

**Supplementary Table S3**

| **S3. Results from Full Multi-Domain Mixed-Effects Model by Domain** | | | |
| --- | --- | --- | --- |
| **Independent Variable** | **β** | **SE** | ***Pseudo-p*** |
| Intercept | -0.646 | 0.318 | 0.043 |
| Time (Years since Baseline) | 0.133 | 0.066 | 0.045 |
| Time2 (Years-since-Baseline, squared) | -0.006 | 0.004 | 0.105 |
| **Need** | | | |
| Baseline HIV Prevalence among PWID per capita | -0.345 | 0.117 | 0.004 |
| Change in HIV among PWID per capita | 0.066 | 0.059 | 0.267 |
| Baseline HIV among PWID per capita * Time | 0.048 | 0.028 | 0.096 |
| Baseline HIV among PWID per capita * Time2 | -0.001 | 0.002 | 0.745 |
| **Resource Availability: General resources** | | | |
| Baseline Percent of Population in Poverty | -0.393 | 0.118 | 0.001 |
| Change in Percent of Population in Poverty | 0.364 | 0.288 | 0.207 |
| Change in Percent of Population in Poverty * Time | -0.069 | 0.048 | 0.148 |
| Change in Percent of Population in Poverty * Time2 | 0.003 | 0.002 | 0.190 |
| Baseline Unemployment Rate | 0.235 | 0.095 | 0.015 |
| Change in Unemployment Rate | 0.365 | 0.134 | 0.007 |
| Change in Unemployment Rate * Time | -0.120 | 0.032 | 0.0002 |
| Change in Unemployment Rate * Time2 | 0.007 | 0.002 | 0.0003 |
| Baseline Median Household Income | -0.005 | 0.104 | 0.958 |
| Change in Median Household Income | 0.212 | 0.361 | 0.557 |
| Change in Median Household Income * Time | -0.053 | 0.057 | 0.348 |
| Change in Median Household Income * Time2 | 0.003 | 0.003 | 0.295 |
| **Resource Availability: Specific resources** | | | |
| Baseline Public Health and Social Work Workforce | 0.322 | 0.090 | 0.001 |
| Change in Public Health and Social Work Workforce | -0.055 | 0.055 | 0.324 |
| Baseline Health Expenditures per Capita | 0.076 | 0.078 | 0.331 |
| Change in Health Expenditures per Capita | -0.030 | 0.056 | 0.590 |
| **Racial structures** | | | |
| Baseline Ratio of Black to White Poverty | -0.116 | 0.074 | 0.119 |
| Change in Ratio of Black to White Poverty | 1.280 | 0.301 | <0.0001 |
| Change in Ratio of Black to White Poverty * Time | -0.175 | 0.051 | 0.001 |
| Change in Ratio of Black to White Poverty * Time2 | 0.007 | 0.002 | 0.006 |
| **Institutional Support: “Liberalism” of public policies** | | | |
| Right-to-Work State (in 1993) | -0.101 | 0.099 | 0.310 |
| **Service Symbiosis** | | | |
| Ever had Syringe Exchange Program (by 1993) | 0.078 | 0.071 | 0.273 |
